# Modelling epidemiological dynamics with pseudo-recovery via fractional-order derivative operator and optimal control measures

**DOI:** 10.1371/journal.pone.0318080

**Published:** 2025-01-30

**Authors:** Samson Olaniyi, Furaha M. Chuma, Ramoshweu S. Lebelo, Richard C. Ogbonna, Sulaimon F. Abimbade

**Affiliations:** 1 Department of Pure and Applied Mathematics, Ladoke Akintola University of Technology, Ogbomoso, Nigeria; 2 Department of Physics, Mathematics and Informatics, Dar es Salaam University College of Education, Dar es Salaam, Tanzania; 3 Department of Applied Physical Sciences, Vaal University of Technology, Vanderbijilpark, South Africa; 4 Department of Computer Science and Mathematics, Evangel University Akaeze, Akaeze, Ebonyi State, Nigeria; Kwame Nkrumah University of Science and Technology, GHANA

## Abstract

In this study, a new deterministic mathematical model based on fractional-order derivative operator that describes the pseudo-recovery dynamics of an epidemiological process is developed. Fractional-order derivative of Caputo type is used to examine the effect of memory in the spread process of infectious diseases with pseudo-recovery. The well-posedness of the model is qualitatively investigated through Banach fixed point theory technique. The spread of the disease in the population is measured by analysing the basic reproduction of the model with respect to its parameters through the sensitivity analysis. Consequently, the analysis is extended to the fractional optimal control model where time-dependent preventive strategy and treatment measure are characterized by Pontryagin’s maximum principle. The resulting Caputo fractional-order optimality system is simulated to understand how both preventive and treatment controls affect the pseudo-recovery dynamics of infectious diseases in the presence of memory. Graphical illustrations are shown to corroborate the qualitative results, and to demonstrate the importance of memory effects in infectious disease modelling. It is shown that time-dependent preventive strategy and treatment measure in the presence of memory engenders significant reduction in the spread of the disease when compared with memoryless situation.

## Introduction

Infectious diseases according to World Health Organization (WHO) [1] and Center for Diseases Control and Prevention (CDC) [2], are disorders caused by microscopic pathogens or harmful agents, such as bacteria, viruses, fungi or parasites. Many of the microscopic organisms live within human bodies. While some are harmless and even helpful, but under certain conditions, they may affect the body system by causing disease. Infectious diseases caused by pathogenic microorganisms can be transmitted through direct or indirect mode of transmission and can also be contracted through the consumption of contaminated food or water or exposure to organisms in the environment [1, 2]. Anyone can be infected with infectious diseases but symptoms may be fatal in individuals with suppressed or compromised immune system, young children, pregnant people, individuals un-vaccinated against infectious diseases, healthcare workers, and human travelers [1]. Serious and life-threatening complications of various infectious diseases are characterized by symptoms such as dehydration, pneumonia, sepsis, meningitis, AIDS and cancer. It has been reported that infectious diseases have claimed lives of numerous human beings and left many vulnerable to the diseases with different adversities [3]. In order to understand and effectively manage the spread dynamics of infectious diseases in the population, mathematical modelling tools can be employed.

Over the years, mathematical modelling, described as simplified representation of abstractions of reality, has been adopted by plethora of researchers to describe and understand the spread dynamics of infectious diseases in human population. See [4–10] and some of the references cited therein on the applications of classical order differential equations to the transmission dynamics of infectious diseases. Notably, one of the classic epidemic models is a simple SIR model [4], which stratifies a homogenous host population into three distinct epidemiological classes namely susceptible, infectious and recovered, of which their population sizes are designated by standard letters *S*, *I*, and *R*, respectively. Till date, the pioneer work of Kermack and McKendrick [4] on the transmission of epidemics in the literature has gained a lot of strong attentions in the mathematical modelling world, where a number of reasonable extensions have been made to gain further insights into the evolution of infectious diseases in human population.

Symptoms of some diseases such as malaria, tuberculosis among others may lead to pseudo-recovery whereby an infectious human who recovered from the disease only acquire transient immunity due to incomplete clearance of the disease in the body system. Pseudo-recovery also called or otherwise known as relapse is a phenomenon whereby dormant clinical symptoms of a disease reappear as a result of incomplete treatment. Unlike the aforementioned mathematical studies in the literature, a few recent studies have been conducted to understand the phenomenon of relapse using mathematical models (see, e.g. [11–19]). The authors in [11] investigated the influence of relapse on the dynamical spread of infectious diseases in human population through the application of mathematical models. The study concluded that efforts should be intensified on the parameters that spontaneously reduce the spread of infectious disease in the population. In [13], a system of nonlinear ordinary differential equations was considered to explore the effect of relapse on the transmission dynamics of malaria taking into account both human and vector populations. Ghosh and co-workers [14] formulated a novel mathematical model to investigate the influence of relapse and reinfection on the transmission dynamics of malaria in human population.

In a related and similar spirit, Abimbade *et al.* [16] stressed on the recurrence of malaria dynamics featuring all the categories of recurrent malaria including relapse. Recently, the complexities associated with the transmission dynamics of different infection cases was studied in [17] purposely to understand the role played by relapse in the spread process. It is not new any longer that the bone of contention to the realization of epidemic-free environment is the recurrence of diseases after total recovery, which in turn leads to the reappearance of symptoms of diseases after treatment. This phenomenon of relapse or pseudo-recovery, being the consequence of incomplete treatment is worth researching into through application of fractional modelling tools.

In view of the foregoing, it is, therefore, imperative to investigate the evolution of infectious diseases with pseudo-recovery taking into account more realistic epidemiological features using fractional order derivative operator. Of note, fractional-order system which generalizes classical order derivative is considered in this study because of its ability to give precise description of real-life situations. More specifically, the peculiarity of fractional order derivative models over classical types is centered on their capacities to capture memory or genetic properties which are essential components of real life situations that cannot be processed by classical order differential equations [20–22]. A number of authors have used the concepts of fractional calculus to describe the influence of memory in various dynamical systems (see, e.g., [23–34] and some of the references cited therein).

In this study, a four dimensional system of equations constituting susceptible, exposed, infectious and recovered humans developed in [35] is fractionalized using fractional-order derivatives of Caputo type. The analysis is mostly centered on the optimal control assessment of the fractional-order epidemiological model, with a view to gaining further insights into the influence of memory on the transmission dynamics of diseases with pseudo-recovery. The remainders of the study are sectionalized as follows. Section is dedicated to the formulation of the fractional-order epidemiological model with pseudo-recovery. The qualitative properties of the model are established in Section. Section presents the analysis of the fractional-order optimal control model with simulations and discussions. While Section wraps up the study with concluding remarks.

## Fractional-order epidemiological model

Here, the Caputo fractional-order non-linear epidemiological model presented to have insightful understanding of the transmission dynamics of infectious diseases is a generalization of the classical-order model studied in [35]. The model characterized by a bilinear incidence function was formulated based on the assumption that incomplete treatment of infection may hinder an infectious individual from attaining permanent immunity against the disease, thereby resulting in pseudo-recovery or relapse. It is important to mention that authors in [35] concentrated on robust stability analysis of the model around both disease-free and endemic equilibria by constructing suitable Lyapunov functionals. Therefore, this generalization is done purposely to examine how the behavior of genetic or memory affect the transmission dynamics of infectious diseases in the population based on optimal control analysis. On this note, it is pertinent to provide some basic concepts of fractional calculus, following the ideas in [28, 29].

**Definition 1** *The Riemann-Liouville fractional ϵ*-*order integral operator of function* $f:R_{+}\to R$, *denoted by* $I_{t}^{\epsilon }f\left(t\right)$ *for t* > 0, *is defined as*

$$\begin{array}{c} I_{t}^{\epsilon }f\left(t\right)=\frac{1}{\Gamma (\epsilon )}\int_{0}^{t}f\left(\varphi \right){\left(t-\varphi \right)}^{\epsilon -1}d\varphi , \end{array}$$ (1)

*where* Γ(*ϵ*), $\epsilon \in R_{+}$ *such that* 0 < *ϵ* ≤ 1, *is the gamma function defined by*

$$\begin{array}{c} \Gamma \left(\epsilon \right)=\int_{0}^{\infty }\vartheta^{\epsilon -1}e^{-\vartheta }d\vartheta . \end{array}$$ (2)

**Definition 2** *The Caputo fractional ϵ*-*order derivative of* $f:R_{+}\to R$, *denoted by* cDtϵf(t), *is defined as*

cDtϵf(t)=1Γ(1-ϵ)∫0tf′(φ)(t-φ)ϵdφ, (3)

*where* $D_{t}^{\epsilon }=d^{\epsilon }/dt^{\epsilon }$.

**Lemma 1** *(Generalized Mean Value Theorem): Let h*(*t*)∈*C*[0, *b*] *and* cDtϵh(t)∈C[0,b] *for* 0 < *ϵ* ≤ 1, *then*

h(t)=h(0)+cDtϵh(ϕ)tϵΓ(ϵ),ϕ∈(0,t),∀t∈(0,b].

*(i) If* cDtϵh(t)≥0 ∀ *t* ∈ (0, *b*), *then h*(*t*) *is non-decreasing*.

*(ii) If* cDtϵh(t)≤0 ∀ *t* ∈ (0, *b*), *then h*(*t*) *is non-increasing*.

**Lemma 2** *Let M*(*t*) ∈ *C*([0, ∞)) *satisfies*

cDtϵM(t)+Aχ(t)≤B,M(0)=M0,

*where ϵ* ∈ (0, 1] *and* A,B∈R *with A* ≠ 0, *then*

$$\begin{array}{c} M\left(t\right)\leq (\chi_{0}-\frac{B}{A})E_{\epsilon ,1}\left(-At^{\epsilon }\right)+\frac{B}{A}, \end{array}$$

*where E*_*ϵ*,1_(⋅) *is a Mittag-Leffler operator given by*

$$\begin{array}{c} E_{\epsilon ,1}\left(x\right)=\sum_{n=0}^{\infty }\frac{x^{n}}{\Gamma (\epsilon n+1)}, \end{array}$$

*which is a generalization of the classical exponential function* $e^{x}=\sum_{n=0}^{\infty }\;x^{n}/n!$, *when ϵ* = 1, *noting that* Γ(*n*) = (*n* − 1)!.

Consequently, the autonomous fractional-order differential equations with bilinear incidence describing the spread dynamics of infectious diseases with pseudo-recovery is given by

cDtϵ(S)(t)=Λ-βS(t)I(t)-μS(t)cDtϵ(E)(t)=βS(t)I(t)-(α+μ)E(t)cDtϵ(I)(t)=αE(t)+θR(t)-(γ+μ)I(t)cDtϵ(R)(t)=γI(t)-(θ+μ)R(t), (4)

with initial conditions

$$\begin{array}{c} S\left(0\right)=S_{0},\;E\left(0\right)=E_{0},\;I\left(0\right)=I_{0},\;R\left(0\right)=R_{0}. \end{array}$$ (5)

The fractional compartmental model (4) splits the total human population, denoted by *N*(*t*), at time *t*, into four mutually exclusive compartments of susceptible individuals represented by *S*(*t*) (population of individuals who are not yet infected by the disease but have the likelihood of contracting the disease); exposed individuals represented by *E*(*t*) (population of individuals who are latently infected with the disease but are incapacitated of transmitting the disease); infectious individuals represented by *I*(*t*) (population of individuals who are clinically infected and are capable of transmitting the disease), and population of pseudo-recovered individuals represented by *R*(*t*) (population of individuals who recovered from the disease with possibility of relapse due to incomplete treatment).

Unlike the classical model [35] which was based on the assumption of constant population size, here, variable population size is assumed. The susceptible population is built up with the recruitment rate of individuals by birth into the population at rate Λ. Susceptible individuals become infected due to their interaction with actively infectious individuals at a bilinear rate *βSI*, where *β* is the effective contact rate. The population of individuals who are latently infected progresses to become actively infected at per-capita rate *α*. The per-capita treatment rate of infectious individuals is denoted by *γ*, while *θ* represents the pseudo-recovery rate of infectious individuals as a result of incomplete treatment. It is important to state that, in accordance with previous studies in the literature (see [29, 36]), the dimensions of the state variables and parameters of the epidemiological model (4) are of fractional (*ϵ*)-order time, *t*^−*ϵ*^.

## Qualitative analysis of the fractional model

In this section, basic fundamental properties of solutions possessed by the non-integer-order epidemiological model (4) are carefully explored.

### Positivity and boundedness

**Theorem 1** *Suppose that the initial conditions S*(0), *E*(0), *I*(0), *R*(0) *are non-negative, the solutions of the fractional-order epidemiological model* (4) *are non-negative for all times*, *t* > 0.

**Proof 1** *It is straightforward from system* (4) *that*

cDtϵ(S)(t)|S=0=Λ≥0,cDtϵ(E)(t)|E=0=βS(t)I(t)≥0,cDtϵ(I)(t)|I=0=αE(t)+θR(t)≥0,cDtϵ(R)(t)|R=0=γI(t)≥0. (6)

*Employing the generalized mean value theorem approach provided in (see, Lemma 1), non-negativity property of the solution of the fractional-order model* (4) *follows, since the vector field’s direction is inward on the bounding planes* $R_{+}^{4}$, *where y* = (*S*, *E*, *I*, *R*), *that is*, cDtϵ(y)(t)|y=0≥0.

The boundedness of solutions, in a region △ defined by

$$\begin{array}{c} △=\{\left(S\left(t\right),E\left(t\right),I\left(t\right),R\left(t\right)\right)\in R_{+}^{4}:N\left(t\right)\leq \frac{\Lambda }{\mu }\}, \end{array}$$ (7)

of the epidemiological model (4) is next explored.

**Theorem 2** *The region* △ *is positively-invariant with respect to the fractional-order epidemiological model* (4).

**Proof 2** *Apparently, the total population of the autonomous fractional-order epidemiological model* (4) *is given by*

cDtϵN(t)=cDtϵS(t)+cDtϵE(t)+cDtϵI(t)+cDtϵR(t)=Λ-μN. (8)

*In what follows by Lemma 2*,

$$\begin{array}{c} N\left(t\right)\leq (N\left(0\right)-\frac{\Lambda }{\mu })E_{\epsilon ,1}(-\mu t^{\epsilon })+\frac{\Lambda }{\mu }. \end{array}$$ (9)

*As t* → ∞, then *N*(*t*) ≤ Λ/*μ*. *Consequently, the solution path of the non-integer-order epidemiological model* (4) *is bounded in the region* △ *by* Λ/*μ*, *insinuating that all solutions initiating in the region △ remains in the region*.

### Existence and uniqueness of solution

In this sub-section, the existence and uniqueness of solutions of the non-integer-order epidemiological model (4) is explored using the Banach’s fixed point theory method [33, 37, 38]. This is done by re-writing model (4) in an initial-valued problem given by

cDtϵ(X(t))=F(t,X(t)),0≤t≤τ,X(0)=X0, (10)

where $X\left(t\right)={\left(S\left(t\right),E\left(t\right),I\left(t\right),R\left(t\right)\right)}^{⊺}$, and $F\left(t,X\left(t\right)\right):\left[0,\tau \right]\times R_{+}^{4}\to R$ defined by $F\left(t,X\left(t\right)\right)={\left(F_{i}\left(t,S,E,I,R\right)\right)}^{⊺}$, *i* = 1, 2, 3, 4, so that

$$\begin{array}{c} \begin{array}{ccc} F_{1}\left(t,S,E,I,R\right) & = & \Lambda -\beta S(t)I(t)-\mu S(t)\\ \;\\ F_{2}\left(t,S,E,I,R\right) & = & \beta S(t)I(t)-(\alpha +\mu )E(t)\\ \;\\ F_{3}\left(t,S,E,I,R\right) & = & \alpha E(t)+\theta R(t)-(\gamma +\mu )I(t)\\ \;\\ F_{4}\left(t,S,E,I,R\right) & = & \gamma I(t)-(\theta +\mu )R(t), \end{array} \end{array}$$ (11)

with $X_{0}={\left(S_{0},E_{0},I_{0},R_{0}\right)}^{⊺}$.

Applying Definition 1 on the initial-valued problem (10) provides

$$\begin{array}{c} X\left(t\right)=X_{0}+\frac{1}{\Gamma (\epsilon )}\int_{0}^{t}{\left(t-\varphi \right)}^{\epsilon -1}F\left(\varphi ,X\left(\varphi \right)\right)d\varphi . \end{array}$$ (12)

Define **D** = (*C*[0, *τ*], ‖⋅‖) as a Banach space for all continuous R-valued functions equipped with the sup-norm described by

$$\begin{array}{c} ∥X\left(t\right)∥=\text{sup}\{|X(t)|:t\in [0,\tau ]\} \end{array}$$

and

$$\begin{array}{c} \text{sup}|X(t)|=\text{sup}(|S(t)|+|E(t)|+|I(t)|+|R(t)|). \end{array}$$

Of particular interest is to demonstrate that F(t,X(t)) satisfies the Lipschitz continuity. This is established as theorized in the next result.

**Theorem 3** F(t,X(t)) *is Lipschitzian in* $C(\left[0,\tau \right]\times R_{+}^{4},R)$ *and t* ∈ [0, *τ*] *for all* $X_{1},X_{2}\in X$ *provided there exists a constant* K>0 *such that*

$$\begin{array}{c} ∥F\left(t,X_{1}\left(t\right)\right)-F\left(t,X_{2}\left(t\right)\right)∥\leq K∥X_{1}\left(t\right)-X_{2}\left(t\right)∥. \end{array}$$ (13)

**Proof 3** *It is essential to bear in mind that the solutions of the fractional-order epidemiological model* (4) *are bounded by* Λ/*μin a positively invariant region △, as proved in Theorem 2. Thereafter, considering* $F_{1}\left(t,S\left(t\right)\right)$ *for S*_1_(*t*) *and S*_2_(*t*), *one sees that*

$$\begin{array}{c} ∥F_{1}\left(t,S_{1}\left(t\right)\right)-F_{1}\left(t,S_{2}\left(t\right)\right)∥\leq ∥\left(\beta I\left(t\right)+\mu \right)∥∥S_{1}-S_{2}∥. \end{array}$$ (14)

*Since I* ≤ Λ/*μ in* △, *then the inequality* (14) *becomes*

$$\begin{array}{c} ∥F_{1}\left(t,S_{1}\left(t\right)\right)-F_{1}\left(t,S_{2}\left(t\right)\right)∥\leq K_{1}∥S_{1}-S_{2}∥, \end{array}$$ (15)

*where* $K_{1}=(\frac{\beta \Lambda }{\mu }+\mu )>0$.

*By similar approach, the following inequalities hold*

$$\begin{array}{c} ∥F_{2}\left(t,E_{1}\left(t\right)\right)-F_{2}\left(t,E_{2}\left(t\right)\right)∥\leq K_{2}∥E_{1}-E_{2}∥, \end{array}$$ (16)

*where* $K_{2}=\left(\alpha +\mu \right)>0.$

$$\begin{array}{c} ∥F_{3}\left(t,I_{1}\left(t\right)\right)-F_{3}\left(t,I_{2}\left(t\right)\right)∥\leq K_{3}∥I_{1}-I_{2}∥, \end{array}$$ (17)

*where* $K_{3}=\left(\gamma +\mu \right)>0$ .

$$\begin{array}{c} ∥F_{4}\left(t,R_{1}\left(t\right)\right)-F_{4}\left(t,R_{2}\left(t\right)\right)∥\leq K_{4}∥R_{1}-R_{2}∥, \end{array}$$ (18)

*where* $K_{4}=\left(\mu +\theta \right)>0$.

*Therefore, condition* (13) *is satisfied, where* $K=\text{max}\{K_{1},K_{2},K_{3},K_{4}\}$ *is the Lipschitz constant*.

Further, define the fixed point of an operator P:D→D by P(X(t))=X(t) so that

$$\begin{array}{c} P\left(X\left(t\right)\right)=X_{0}+\frac{1}{\Gamma (\epsilon )}\int_{0}^{t}{\left(t-\varphi \right)}^{\epsilon -1}P\left(\varphi ,X\left(\varphi \right)\right)d\varphi . \end{array}$$ (19)

The next result is claimed.

**Theorem 4** *The non-integer-order epidemiological model* (4) *possesses a unique solution provided that* $\tau^{\epsilon }K/\epsilon \Gamma \left(\epsilon \right)<1$.

**Proof 4** *The proof is based on demonstrating that* P *is a contraction. Since* F(t,X(t)) *is Lipschitz continuous, then for* $X_{1}\left(t\right),X_{2}\left(t\right)\in D$ *and* 0 ≤ *t* ≤ *τ*, *it then follows that*

$$\begin{array}{c} \begin{array}{ccc} ∥P\left(X_{1}\left(t\right)\right)-P\left(X_{2}\left(t\right)\right)∥ & = & ∥\frac{1}{\Gamma (\epsilon )}\int_{0}^{t}{\left(t-\varphi \right)}^{\epsilon -1}[F\left(\varphi ,X_{1}\left(\varphi \right)\right)-F\left(\varphi ,X_{2}\left(\varphi \right)\right)]d\varphi ∥\\ \;\\ & \leq & \frac{1}{\Gamma (\epsilon )}\int_{0}^{t}{\left(t-\varphi \right)}^{\epsilon -1}∥F\left(\varphi ,X_{1}\left(\varphi \right)\right)-F\left(\varphi ,X_{2}\left(\varphi \right)\right)∥d\varphi \\ \;\\ & \leq & \frac{K}{\Gamma (\epsilon )}∥X_{1}\left(t\right)-X_{2}\left(t\right)∥\int_{0}^{t}{\left(t-\varphi \right)}^{\epsilon -1}d\varphi \\ \;\\ & \leq & \frac{\tau^{\epsilon }K}{\epsilon \Gamma (\epsilon )}∥X_{1}\left(t\right)-X_{2}\left(t\right)∥, \end{array} \end{array}$$ (20)

*It therefore implies that* P *is a contraction, whenever* $\tau^{\epsilon }K/\epsilon \Gamma \left(\epsilon \right)<1$ . *Thus, the fractional-order epidemiological model* (4) *has a unique solution*.

### Basic reproduction number of the model

Here in this subsection, the epidemiological threshold that measures the spread potential of infectious diseases is examined through the implementation of the famous next generation matrix approach [39]. Since the basic reproduction number of the fractional order model depends on the disease-free equilibrium point of the model, it worth noting that the equilibrium points and its stability analysis are as obatined in [35]. By definition, basic reproduction number, denoted by $R_{0}$, is the number of secondary cases of infection produced by a single infectious individual during its period of infectiousness in a completely naive population. Using the next generation matrix approach, $R_{0}=\rho \left(FV^{-1}\right)$, where the matrices *F* and *V* representing transmission and transitions terms of the model are given, respectively, by

$$\begin{array}{c} F=(\begin{array}{ccc} 0 & \frac{\beta \Lambda }{\mu } & 0\\ 0 & 0 & 0\\ 0 & 0 & 0 \end{array})\;\text{and}\;\;V=(\begin{array}{ccc} \alpha +\mu & 0 & 0\\ -\alpha & \gamma +\mu & -\theta \\ 0 & -\gamma & \theta +\mu \end{array}). \end{array}$$

As a consequence, the basic reproduction number of the model (4) is obtained as

$$\begin{array}{c} R_{0}=\frac{\alpha \beta \Lambda (\theta +\mu )}{\mu^{2}\left(\alpha +\mu \right)\left(\theta +\gamma +\mu \right)}. \end{array}$$ (21)

**Fig 1:**
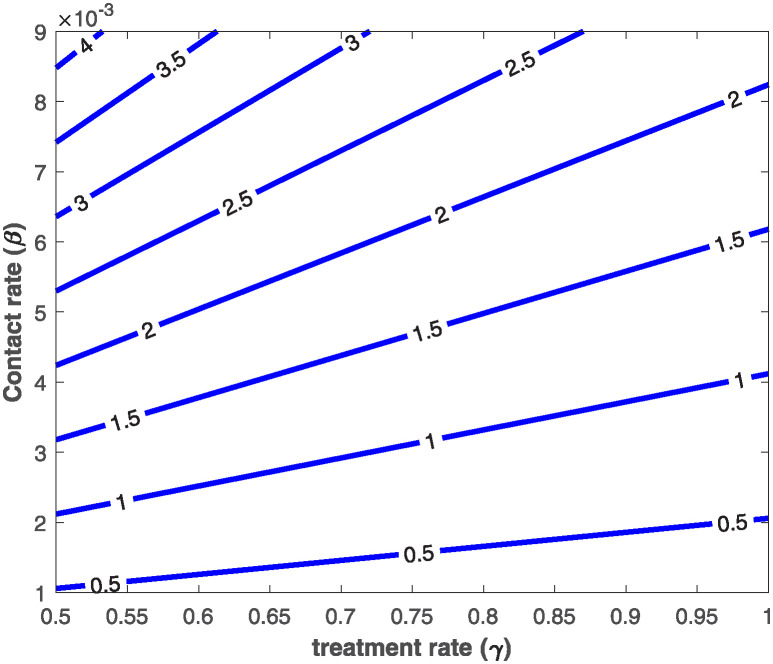
2-D contour plot showing how effective contact rate, *β*, and treatment rate, *γ*, affect the value of the basic reproduction number, $R_{0}$. The values of the other parameters are chosen as Λ = 10, *α* = 0.01, *θ* = 0.01, *μ* = 0.02.

**Fig 2:**
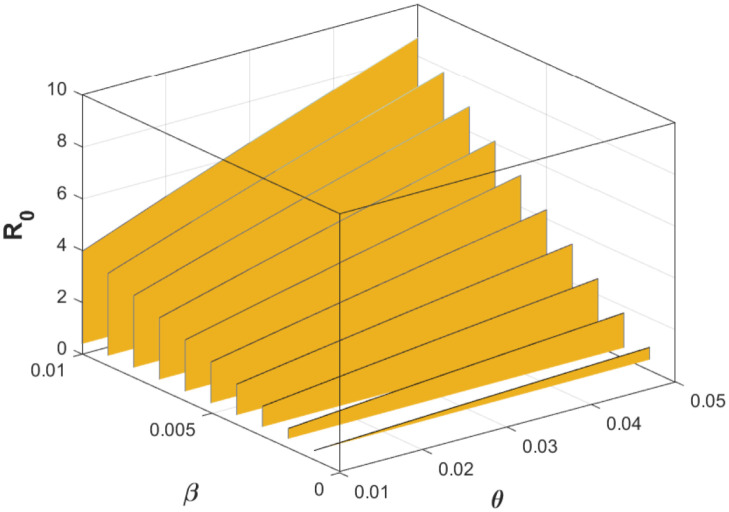
3-D contour plot showing how effective contact rate, *β*, and pseudo-recovery rate, *θ*, affect the value of the basic reproduction number, $R_{0}$. The values of the other parameters are chosen as Λ = 10, *α* = 0.01, *γ* = 0.6, *μ* = 0.02.

It is imperative to expatiate that this epidemiological threshold, $R_{0}$, is important in setting certain preventive or control measures that will allow for effective containment of infectious diseases. As displayed in the Fig 1, one can see that unhindered increase in the effective contact rate *β* leads to an increase in the basic reproduction number, $R_{0}$, from a disease-free state to an endemic state. While increase in treatment rate, *γ*, reduces the value of the basic reproduction number. In another perspective, Fig 2 confirms how increase in effective contact rate contributes to a surge in the epidemiological threshold. Similarly, as pseudo-recovery rate increases, it can be seen that the basic reproduction number increases to a very endemic state. These results are evident in the fact that the basic reproduction number, $R_{0}$, is an increasing function of both effective contact and pseudo-recovery rates, while $R_{0}$ is a decreasing function of the treatment rate. Specifically, it is easy to see that

$$\begin{array}{c} \begin{array}{cc} \frac{\partial R_{0}}{\partial \beta }= & \frac{\alpha \Lambda (\theta +\mu )}{\mu^{2}\left(\alpha +\mu \right)\left(\gamma +\mu +\theta \right)}>0,\\ \;\frac{\partial R_{0}}{\partial \theta }= & \frac{\alpha \beta \Lambda \gamma }{\mu^{2}\left(\alpha +\mu \right){\left(\gamma +\mu +\theta \right)}^{2}}>0,\\ \;\frac{\partial R_{0}}{\partial \gamma }= & -(\frac{\alpha \beta \Lambda (\theta +\mu )}{\mu^{2}\left(\alpha +\mu \right){\left(\gamma +\mu +\theta \right)}^{2}})<0. \end{array} \end{array}$$

To this end, an optimal control model incorporating time-dependent optimal control functions to hinder the spread of infectious disease with pseudo-recovery is presented in the next section.

## Fractional-order optimal control model

The non-autonomous version of the fractional-order epidemiological model with pseudo-recovery is given by

cDtϵ(S)(t)=Λ-(1-u1(t))βS(t)I(t)-μS(t)cDtϵ(E)(t)=(1-u1(t))βS(t)I(t)-(α+μ)E(t)cDtϵ(I)(t)=αE(t)+θR(t)-(γ+ku2(t)+μ)I(t)cDtϵ(R)(t)=(γ+ku2(t))I(t)-(θ+μ)R(t), (22)

with the control function *u*_1_(*t*) being the control responsible for preventing effective contact of infectious diseases. This control may serve as public awareness against the disease. The control *u*_2_(*t*) is responsible for treatment of infectious individuals to enhance recovery. The cost functional required to minimize the spread of infectious diseases as well as minimizing the associated costs of implementing the control functions of the fractional-order model is given by

$$\begin{array}{c} J=\int_{0}^{t_{f}}(C_{0}I\left(t\right)+\frac{1}{2}\sum_{i=1}^{2}B_{i}u_{i}^{2})dt, \end{array}$$ (23)

where *t*_*f*_ is the stipulated window period required to minimize the objective functional and *C*_0_ is the positive balancing weight constant. While $B_{1}u_{1}^{2}$ and $B_{2}u_{2}^{2}$ are, respectively, the implementation costs for the controls *u*_1_ and *u*_2_. It is worth mentioning that *u*_*i*_ ∈ [0, 1], *i* = 1, 2, with *u*_*i*_ = 0 being a situation where no control effort is put in place to contain the spread of infectious diseases, and *u*_*i*_ = 1 on the other hand denotes the maximum control effort implemented in minimizing the disease in the population. More importantly, it should be noted that the quadratic nature of the cost functions allowing for explicit derivation of the control functions are in agreement with the existing studies on optimal control problems in the literature [40–43]. In particular, the choice of minimizing the objective functional (23) is centered on finding an optimal control double $u^{*}=\left(u_{1}^{*},u_{2}^{*}\right)$, such that

$$\begin{array}{c} J\left(u^{*}\right)=\text{min}\left\{J\left(u_{i}\right):u_{i}\in U\right\}, \end{array}$$ (24)

where J is a Lebesgue measurable control set given by

$$\begin{array}{c} U=\{u_{i}:0\leq {u_{i}}_{min}\leq u_{i}\left(t\right)\leq {u_{i}}_{max}\leq 1,\;0\leq t\leq t_{f}\}. \end{array}$$ (25)

### Existence of optimal control double

Next is to establish the existence of the optimal control double u* satisfying the minimization problem specified in (24) subject to the fractional state system (22). This is done by speculating an existence result given by.

**Theorem 5** There exists an optimal control double u* *satisfying* $J\left(u^{*}\right)=\text{min}\left\{J\left(u_{1},u_{2}\right):\left(u_{1},u_{2}\right)\in U\right\}$ *subject to the fractional-order state system* (22).

The proof of the existence Theorem 5 is due to the preservation of the following properties (see, [44–46]):

(P1). The control set U is convex and closed.
(P2). The fractional-order state system (6) is bounded by a linear function in both state and control variables.
(P3). The Lagrangian of the objective functional is convex with respect to the optimal control double.
(P4). There exist constants *a*_1_, *a*_2_ > 0 and *a*_3_ > 1 such that the Lagrangian is bounded below by $a_{1}\left(\right|u_{i}{{|}^{2})}^{\frac{a_{3}}{2}}-a_{2}$, *i* = 1, 2.

**Proof 5** *Each of the properties (P1)–(P4) is established as follows*.

*(P1). It can easily be deduced from the control set* (25) *that the set* U *is closed by definition. Additionally, for any two arbitrary points* p,q∈U, *with p* = (*p*_1_, *p*_2_) *and q* = (*q*_1_, *q*_2_). *It follows that*

$$\begin{array}{c} \left(\vartheta p_{i}+\left(1-\vartheta \right)q_{i}\right)\in {\left[0,1\right]}^{2},\forall \;\vartheta \in \left[0,1\right],i=1,2. \end{array}$$

*Then*, (ϑp+(1-ϑ)q∈U), *which satisfies the definition of a convex set* [47].

*(P2). Using the boundedness of the state variables of the epidemiological model* (4) *given in Theorem 2, the fractional order epidemiological model* (22) *can be written as a linear function of the control v* = (*u*_1_, *u*_2_) *with time and state variables-dependent coefficients, as explicitly proved in* [40]. *As a result, it can be established that the right-hand side of the fractional-order epidemiological model* (22) *is bounded above by a sum of bounded state and control*.

*(P3). The Lagrangian mostly regarded as the integrand of the cost functional is given by*

$$\begin{array}{c} L\left(t,x,v\right)=C_{0}I\left(t\right)+\frac{1}{2}\sum_{i=1}^{2}B_{i}u_{i}^{2}, \end{array}$$ (26)

*where x* = (*S*, *E*, *I*, *R*) *and v* = (*u*_1_, *u*_2_). *Now*, ∀ $p=(p_{1},p_{2})\in U$ *and* $q=(q_{1},q_{2})\in U$, *with ϑ belonging to* [0, 1]. *Then, it follows from* (26) *that*

$$\begin{array}{c} L\left(t,x,\vartheta p+\left(1-\vartheta \right)q\right)=C_{0}I\left(t\right)+\frac{1}{2}\sum_{i=1}^{2}B_{i}{\left(\vartheta p_{i}+\left(1-\vartheta \right)q_{i}\right)}^{2}, \end{array}$$

*while*

$$\begin{array}{c} \vartheta L\left(t,x,p\right)+\left(1-\vartheta \right)L\left(t,x,q\right)=C_{0}I\left(t\right)+\frac{1}{2}\vartheta \sum_{i=1}^{2}B_{i}p_{i}^{2}+\frac{1}{2}\left(1-\vartheta \right)\sum_{i=1}^{2}B_{i}q_{i}^{2}. \end{array}$$

*Consequently*,

$$\begin{array}{c} L\left(t,x,\vartheta p+\left(1-\vartheta \right)q\right)-\left(\vartheta L\left(t,x,p\right)+\left(1-\vartheta \right)L\left(t,x,q\right)\right)=\frac{1}{2}\left(\vartheta^{2}-\vartheta \right)\sum_{i=1}^{2}B_{i}{\left(p_{i}-q_{i}\right)}^{2}. \end{array}$$ (27)

*Since* 0 ≤ *ϑ* ≤ 1, *it is clear from* (27) *that*

$$\begin{array}{c} L(t,x,\vartheta p+(1-\vartheta )q)\leq \vartheta L(t,x,p)+(1-\vartheta )L(t,x,q). \end{array}$$ (28)

*Therefore, the Lagrangian is a convex function*.

*(P4). It is straightforward from the Lagrangian* (26), *that*

$$\begin{array}{c} L\left(t,x,v\right)\geq a_{1}{\left(\sum_{i=1}^{2}u_{i}^{2}\right)}^{\frac{a_{3}}{2}}-a_{2}, \end{array}$$ (29)

*where a*_1_ = min{*B*_1_/2, *B*_2_/2}, *a*_2_ ≥ 0 *and a*_3_ = 2. *This completes the proof of the existence of an optimal control double*.

### Characterization of optimal control double

Here, the two control functions *u*_1_(*t*) and *u*_2_(*t*) are characterized by restructuring the minimization problem described in (24) into an auxiliary problem of minimizing pointwise, a Hamiltonian H subject to the control functions. This goal is achieved by utilizing the famous Pontryagin’s maximum principle [48]. The Hamiltonian required for the optimal control problem is given by

$$\begin{array}{c} \begin{array}{cc} H= & C_{0}I\left(t\right)+B_{1}u_{1}^{2}\left(t\right)+B_{2}u_{2}^{2}\left(t\right)\\ \;\\ & +\lambda_{1}(\Lambda -(1-u_{1}\left(t\right))\beta S\left(t\right)I\left(t\right)-\mu S\left(t\right))\\ \;\\ & +\lambda_{2}((1-u_{1}\left(t\right))\beta S\left(t\right)I\left(t\right)-\left(\alpha +\mu \right)E\left(t\right))\\ \;\\ & +\lambda_{3}(\alpha E\left(t\right)+\theta R\left(t\right)-(\gamma +ku_{2}\left(t\right)+\mu )I\left(t\right))\\ \;\\ & +\lambda_{4}((\gamma +ku_{2}\left(t\right))I\left(t\right)-\left(\theta +\mu \right)R\left(t\right)), \end{array} \end{array}$$ (30)

where λ_1_, λ_2_, λ_3_ and λ_4_ are the adjoint variables associated with the state variables of the time-variant fractional-order epidemiological model. Then, the next result is established

**Theorem 6** *Given an optimal control double* $\left(u_{1}^{*},u_{2}^{*}\right)$ *minimizing the objective functional* (23) *over the control set* U, *and subject to the non-autonomous fractional order epidemiological model* (22), *then there exist adjoint variables* λ_1_, λ_2_, λ_3_ *and* λ_4_, *that satisfy the adjoint system given by*

cDtϵλ1=(λ1-λ2)(1-u1(t))βI+μλ1,cDtϵλ2=(λ2-λ3)α+μλ2,cDtϵλ3=(λ1-λ2)(1-u1(t))βS+μλ3+(λ3-λ4)(γ+ku2(t))-A,cDtϵλ4=(λ4-λ3)θ+μλ4, (31)

*with the transversality conditions*

$$\begin{array}{c} \;\lambda_{x}\left(t_{f}\right)=0,\;\;\;x=\left(S,E,I,R\right), \end{array}$$ (32)

*and the optimal control double characterizations*

$$\begin{array}{c} \begin{array}{cc} u_{1}^{*}= & \text{max}\{0,\text{min}\{\frac{\beta SI(\lambda_{2}-\lambda_{1})}{2B_{1}},1\}\},\\ \;\\ u_{2}^{*}= & \text{max}\{0,\text{min}\{\frac{kI(\lambda_{3}-\lambda_{4})}{2B_{2}},1\}\}.\\ \; \end{array} \end{array}$$ (33)

**Proof 6** *The proof is established by taking the partial derivatives of the Hamiltonian* H (30) *with respect to each of the state variables of the fractional-order model to obtain the adjoint system given in* (31). *This is done as follows*

cDtϵλx=-∂H∂x,λx(tf)=0,wherex=(S,E,I,R).

*In addition, the optimal control characterizations* (33) *can be attained by solving for* $u_{1}^{*}$ *and* $u_{2}^{*}$, *respectively, from the optimality condition*

$$\begin{array}{c} \begin{array}{c} \frac{\partial H}{\partial u_{1}}=2B_{1}u_{1}^{*}-\beta SI\left(\lambda_{2}-\lambda_{1}\right)=0,\\ \;\\ \frac{\partial H}{\partial u_{2}}=2B_{2}u_{2}^{*}-kI\left(\lambda_{3}-\lambda_{4}\right)=0. \end{array} \end{array}$$ (34)

*It then follows by standard control arguments involving bounds that*

$$\begin{array}{c} u_{i}^{*}=\{\begin{array}{cc} 0, & for\;\varphi_{i}^{*}\leq 0\\ \varphi_{i}^{*}, & for\;0<\varphi_{i}^{*}<1\\ 1, & for\;\varphi_{i}^{*}\geq 1, \end{array} \end{array}$$

*for i* = 1, 2 *and where*

$$\begin{array}{c} \begin{array}{ccc} \varphi_{1}^{*} & = & \frac{\beta SI(\lambda_{2}-\lambda_{1})}{2B_{1}},\\ \;\\ \varphi_{2}^{*} & = & \frac{kI(\lambda_{3}-\lambda_{4})}{2B_{2}}. \end{array} \end{array}$$ (35)

*This completes the proof*.

### Simulations

**Fig 3:**
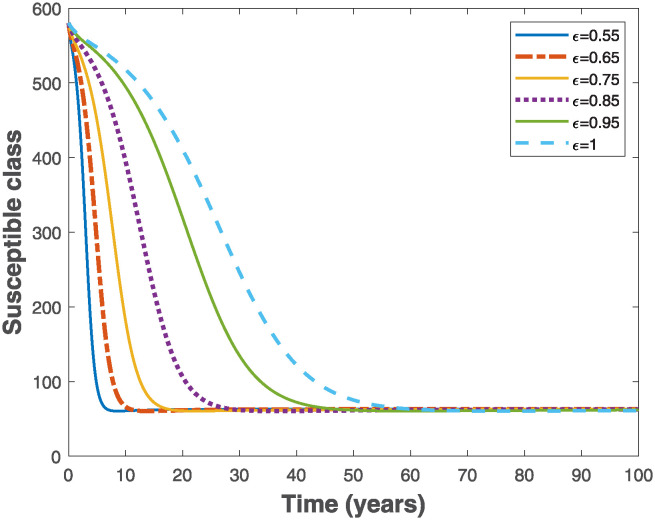
Behaviours of the trajectories of the susceptible populations of the fractional-order epidemiological model (4) by varying the fractional order, *ϵ* using the parameter values Λ = 10, *β* = 0.02, *α* = 0.01, *θ* = 0.01, *γ* = 0.6 and *μ* = 0.02, so that $R_{0}=7.9365>1$.

**Fig 4:**
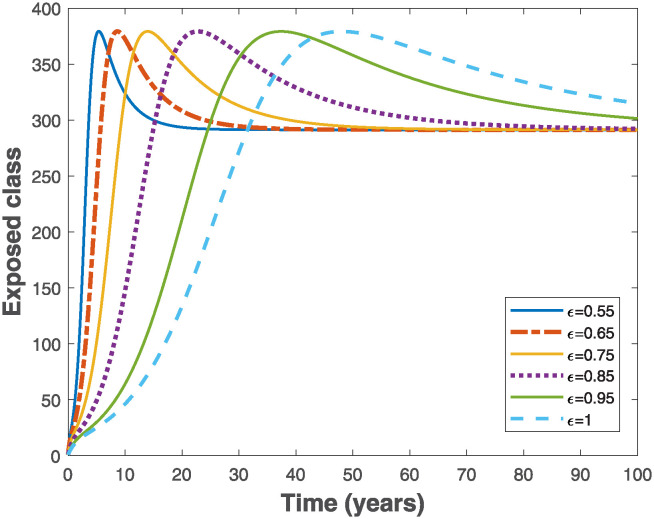
Behaviours of trajectories of exposed population of the fractional-order epidemiological model (4) by varying the fractional order, *ϵ* using the parameter values Λ = 10, *β* = 0.02, *α* = 0.01, *θ* = 0.01, *γ* = 0.6 and *μ* = 0.02, so that $R_{0}=7.9365>1$.

**Fig 5:**
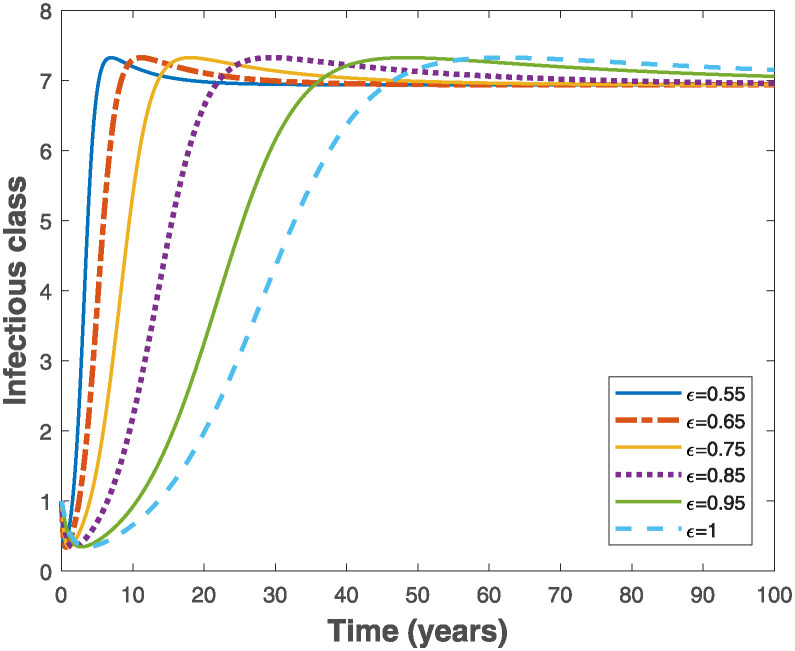
Behaviours of the trajectories of infectious population of the fractional-order epidemiological model (4) by varying the fractional order, *ϵ* using the parameter values Λ = 10, *β* = 0.02, *α* = 0.01, *θ* = 0.01, *γ* = 0.6 and *μ* = 0.02, so that $R_{0}=7.9365>1$.

**Fig 6:**
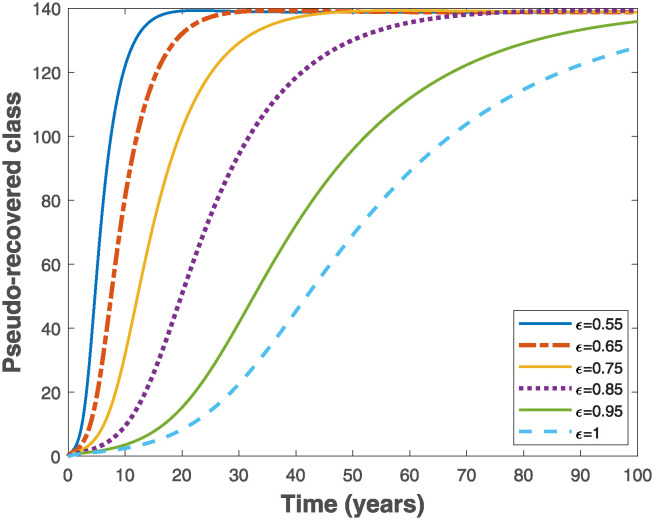
Behaviours of the trajectories of pseudo-recovered class of the fractional-order epidemiological model (4) by varying the fractional order, *ϵ* using the parameter values Λ = 10, *β* = 0.02, *α* = 0.01, *θ* = 0.01, *γ* = 0.6 and *μ* = 0.02, so that $R_{0}=7.9365>1$.

Fractional Euler’s method provided in [49] is used to simulate the fractional-order model (4) in order to visualize the importance of memory effects on the behaviour of solutions of the system. As the fractional order varies in the interval 0 < *ϵ* ≤ 1, it is observed that the trajectories of susceptible and exposed populations in Figs 3 and 4, respectively converge faster with reduced fractional order. Similar behaviours are observed in Figs 5 and 6 for the trajectories of infectious and pseudo-recovered classes, respectively. Precisely, this result shows that presence of memory (i.e., when *ϵ* < 1) makes the control of infectious disease easier than a memoryless case where *ϵ* = 1. Moreover, simulations of the fractional-order optimality system comprising both fractional-order state model (22) and adjoint system (31) with their corresponding initial and transversality conditions are solved simultaneously with the optimal characterizations (33) using the generalized Euler’s forward and backward sweep method programmed in MATLAB [21]. The final time is taken to be *t*_*f*_ ∈ [0, 10] measured in years, while the weight constants *C*_0_ = 1, *B*_1_ = 1 and *B*_2_ = 0.5 are used for the simulations when the rate constant *k* = 1.

**Fig 7:**
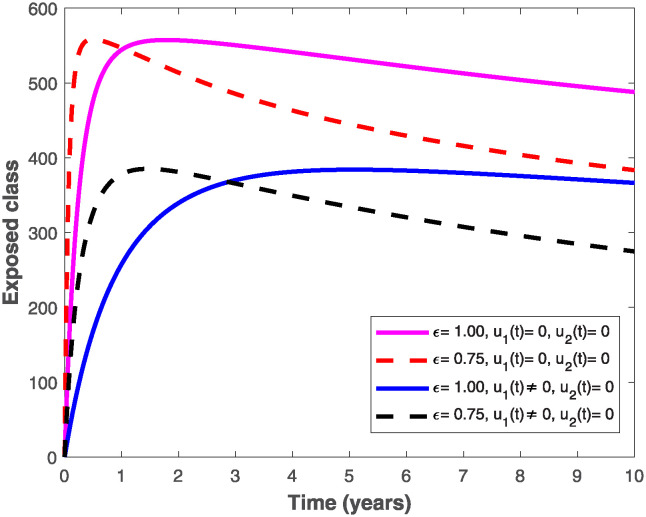
Behaviour of the exposed population by implementing the optimal preventive control, *u*_1_(*t*), with and without memory.

**Fig 8:**
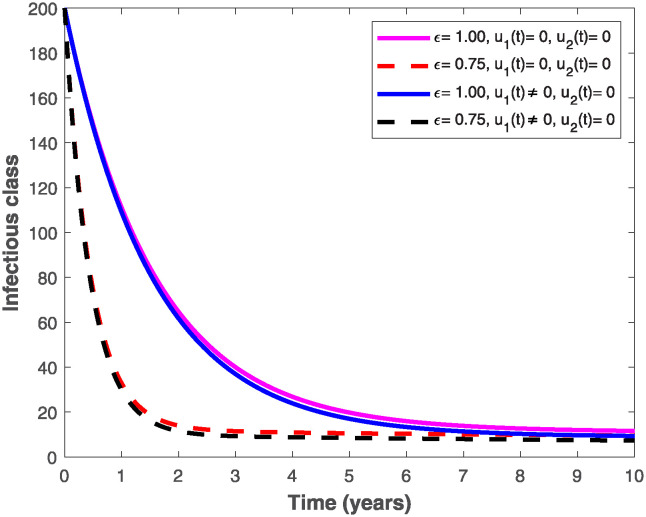
Behaviour of the infectious population by implementing the optimal preventive control, *u*_1_(*t*), with and without memory.

**Fig 9:**
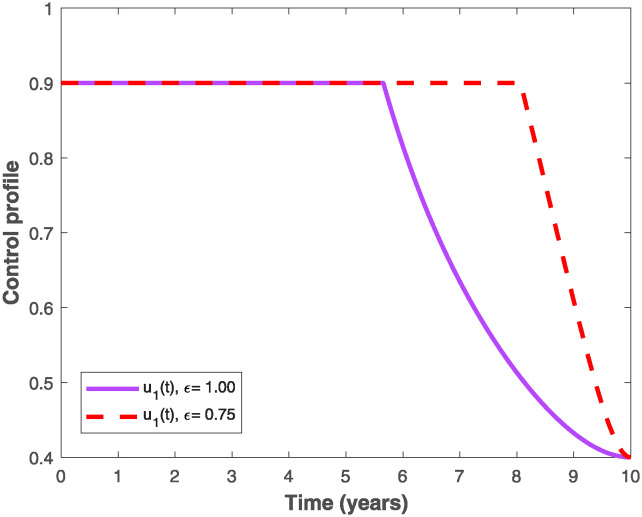
Optimal control profile for implementing the preventive control, *u*_1_(*t*), with and without memory.

**Fig 10:**
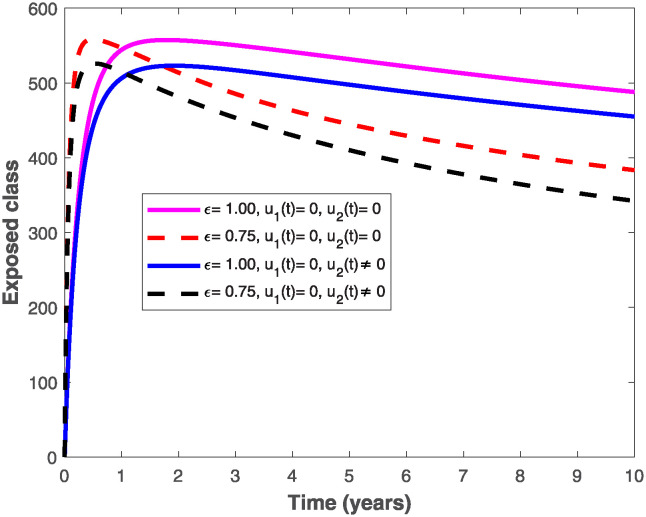
Behaviour of the exposed population by implementing the optimal treatment control, *u*_2_(*t*), with and without memory.

**Fig 11:**
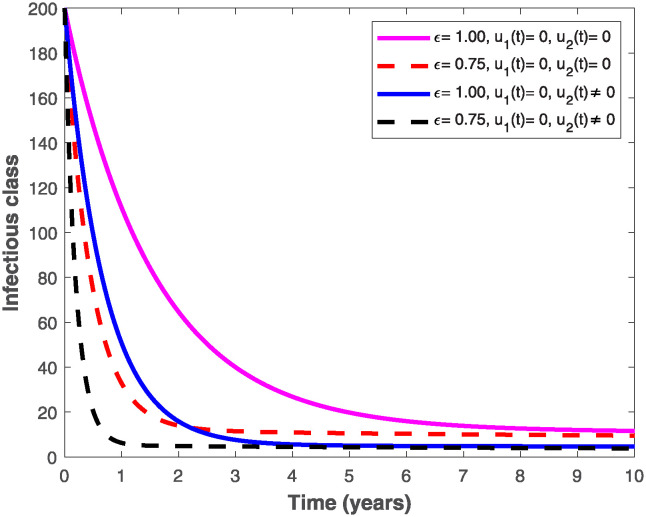
Behaviour of the infectious population by implementing the optimal treatment control, *u*_2_(*t*), with and without memory.

**Fig 12:**
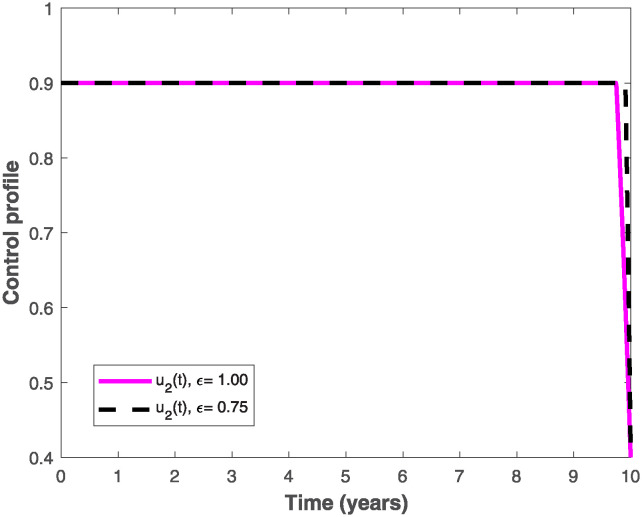
Optimal control profile for implementing the treatment control, *u*_2_(*t*), with and without memory.

**Fig 13:**
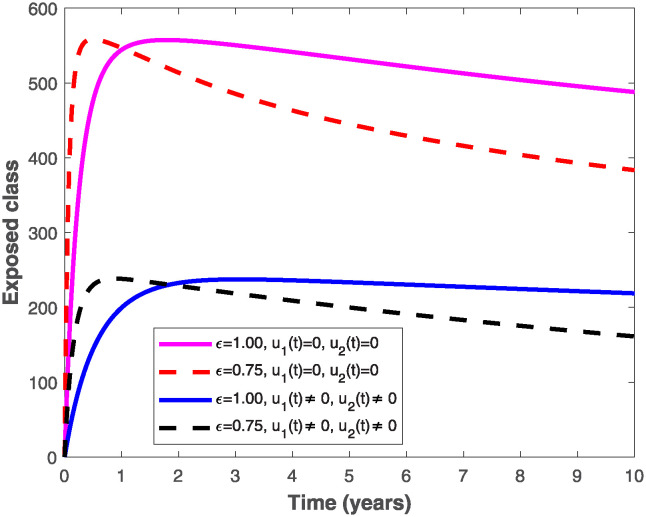
Behaviour of the exposed population by implementing both optimal preventive and treatment controls, *u*_1_(*t*) *and u*_2_(*t*), with and without memory.

**Fig 14:**
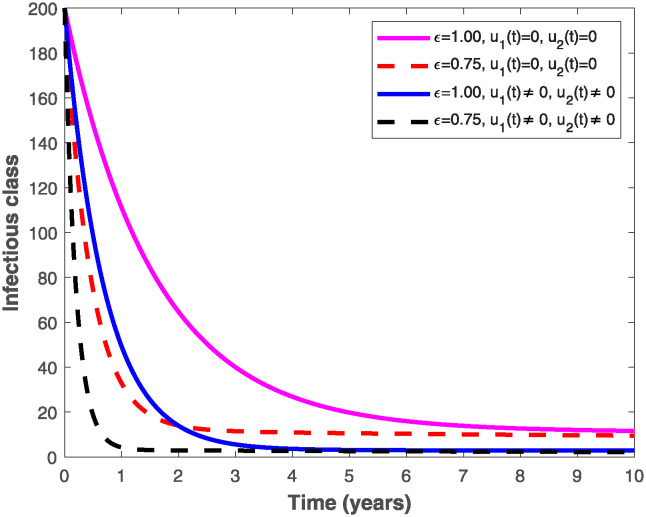
Behaviours of the infectious population by implementing both optimal preventive and treatment controls, *u*_1_(*t*) *and u*_2_(*t*), with and without memory.

**Fig 15:**
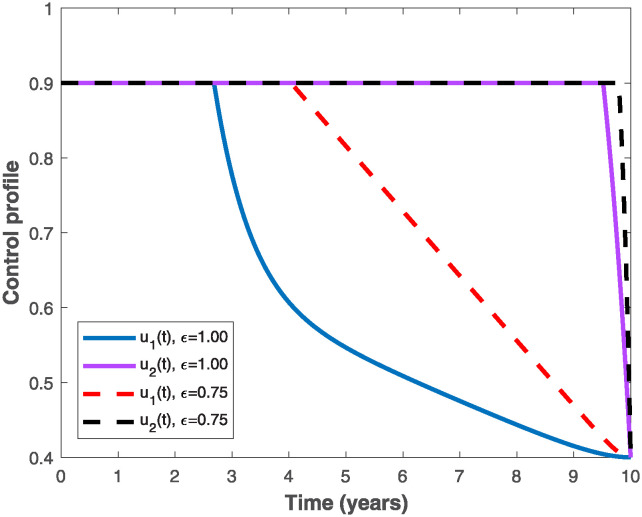
Optimal control profile for implementing both optimal preventive and treatment controls, *u*_1_(*t*) *and u*_2_(*t*), with and without memory.

The populations of exposed and infected individuals shown in Figs 7 and 8, respectively decrease mostly when the preventive control *u*_1_(*t*) shown in Fig 9 is optimally implemented at 90% in the presence of memory with *ϵ* = 0.75. It is interesting to note that the infectious disease is more prevalent without memory (i.e., when *ϵ* = 1), even in the presence of the optimal preventive control. This confirms the great influence of memory effects in controlling the spread of infectious diseases in the population. Similarly, the populations of exposed and infectious individuals reduce mostly in the presence of memory with *ϵ* = 0.75 as shown in Figs 10 and 11 when single treatment control *u*_2_(*t*) given in Fig 12 is at optimal level. As expected, in comparison with the implementation of each of the optimal controls, combination of both controls (*u*_1_(*t*) and *u*_2_(*t*)) with memory reduces the sizes of exposed and infectious individuals significantly as depicted in Figs 13 and 14, respectively. The control profile describing the optimal implementation of the double control is provided in Fig 15.

The implication of this from the epidemiological view point is that, application of fractional calculus in modelling the dynamics of infectious diseases with pseudo-recovery gives a precise and accurate description of the time evolution of infectious diseases compared to its classical order counterparts (i.e., when *α* = 1). This great feature is due to the presence of the non-integer order in the fractional model (i.e., when *α* < 1), which can be used as a fit parameter for hindering the emergence of infectious diseases in the population.

## Conclusion

With a view to providing further insights into modelling of infectious diseases with pseudo-recovery due to incomplete treatment, a mathematical model which generalizes the existing constant population classical model has been developed in this work. A Caputo fractional-order derivative operator, which has ability to describe dynamics of real-world systems more accurately due to its inherent nature of capturing memory or hereditary properties, was used to formulate the epidemiological model in the presence of pseudo-recovery phenomenon. Rigorous analysis through Banach fixed point theory was performed to establish the well-posedness of the model. Particularly, existence and uniqueness of solutions of the fractional-order epidemiological model was proved. The next generation matrix approach was used to find the basic reproduction number—an important threshold quantity that informs whether an infectious disease will be prevalent or cease to exist in the population. Interestingly, the basic reproduction number captured all the six parameters of the fractional-order model, enabling robust assessment of the threshold quantity with respect to all the parameters. Specifically, it was revealed that the threshold quantity is an increasing function of effective contact rate and pseudo-recovery rate of the model, while the threshold quantity is a decreasing function of the treatment rate. With this insight, a time-variant fractional-order epidemiological model was considered to incorporate optimal prevention and treatment measures. The existence of the two optimal control functions was proved, and characterizations of the controls were done by using Pontryagin’s maximum principle.

Simulations carried out to consolidate the analysis in this study showed that while the presence of memory due to fractional-oder derivative operator could help in controlling the transmission of infectious diseases in the population, however, most desirable disease control could be achieved when both time-dependent optimal prevention and treatment measures are implemented simultaneously in the presence of memory. Therefore, it can be inferred that the combination of memory effects and optimal control is a good synergy for curbing the spread of infectious diseases in a most desirable way. In additon, it should be noted that the formulated model is generic for all infectious diseases that exhibit pseudo-recovery property. For this reason, parameters used in simulating the deterministic fractional-order model were hypothetically chosen. However, real-world datasets for a specific disease could be used to fit the model in order to demonstrate its applicability. Therefore, future study might consider stochastic version of the fractional-order model in order to capture the randomness or fluctuations that may arise in transmission of any specific disease with pseudo-recovery using real-world datasets.
